# Selective hydrogenation of vanillin over a graphene-encapsulated nitrogen-doped bimetallic magnetic Ni/Fe@NDC nano-catalyst

**DOI:** 10.1039/d4ra02729a

**Published:** 2024-05-23

**Authors:** Siyi Mi, Lungang Chen, Xinghua Zhang, Qi Zhang, Longlong Ma, Jianguo Liu

**Affiliations:** a Southeast University-Monash University Suzhou Joint Graduate School, Southeast University Suzhou 215000 China; b School of Energy and Environment, Southeast University Nanjing 210096 China liujg@seu.edu.cn

## Abstract

One of the main obstacles to the development of sustainable biomass feedstocks today is the research of selective hydrogenation of biomass platform compounds for the synthesis of high-value chemicals. This work reports on the synthesis of a Ni/Fe bimetallic catalyst with nitrogen-doped carbon serving as the carrier, hydrogen serving as the primary donor, and isopropanol serving as the reaction medium and serving as a secondary donor. Vanillin was catalytically hydrogenated to produce 4-methylguaiacol, a complete hydrogenation product, under a reaction temperature of 200 °C for four hours. A single product with a good yield (95.26% conversion and selectivity up to 99%) was achieved by the moderate conditions, offering a potential route for the catalytic hydrogenation of biomass platform compounds.

## Abstract 2

One of the main obstacles to the development of sustainable biomass feedstocks today is the research of selective hydrogenation of biomass platform compounds for the synthesis of high-value chemicals.

## Graphical abstract

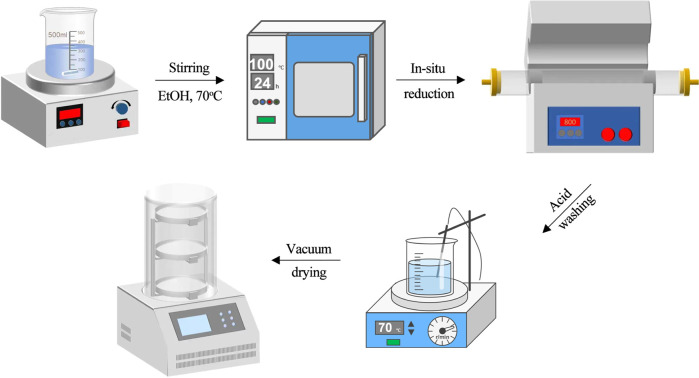

## 1. Introduction

Vanillin, a key ingredient in pyrolysis oil made from lignin fractions, is a promising biomass platform chemical produced by thermally catalyzing the depolymerization of lignin biomass.^1^ The three types of oxygen-containing functional groups found in this species – hydroxyl, ether, and aldehyde groups – can be hydrogenated selectively to produce vanillyl alcohol (VA) and 2-methoxy-4-methylphenol (MMP), the latter of which has the potential to be a future biofuel and is currently used extensively in the production of pharmaceutical intermediates and spices.^2^ As a result, MMP is frequently used as a model compound in studies examining the selective conversion of –CHO to –CH_3_.^3^ Among them, catalysts based on noble metals, such as Pd, Pt, Rh, Ru, *etc.*, are frequently employed in this kind of reaction and exhibit superior catalytic effects.^4^

To create a novel low-loading Pd/CNS catalyst that would enable selective hydrogenation of vanillin in moderate circumstances, Kuang *et al.*^5^ utilized N,S-doped carbon spheres as carriers. Zhang *et al.*^6^ revealed that the Pd catalyst supported by ZrO_2_ exhibited remarkable efficiency in the vanillin hydrodeoxygenation reaction, offering suggestions for turning bio-oil into environmentally friendly fuels. Pd nanoparticles are extremely effective at hydrogenating carbonyl groups and then hydrogenolysis alcohol bonds,^7^ this is especially true when the particles are incorporated into carbon-based materials.^8^ However, because precious metals have a strong hydrogenation capacity, it is simple to over-hydrogenate the benzene ring or to trigger unfavorable side reactions such as hydroxyl hydrogenolysis and ether bond cleavage.^9^ There are several drawbacks to the depletion of active metals and finite reserves of precious metals, including low utilization and high expense. There are a lot of problems with using it for catalytic hydrogenation.^10^

Following this, non-noble metal catalysts supported on a variety of carriers exhibit significant utility value, including metal oxides, porous carbons, and zeolites.^11^ According to Li *et al.*,^12^ catalysts supported on hierarchical porous carbon without nitrogen doping exhibited lower catalytic activity than Ni nanoparticles well distributed on nitrogen-doped hierarchical porous carbon. Similarly, cobalt nanoparticles placed on nitrogen-doped carbon were found by Jiang *et al.*^13^ to have good activity and stability for the reduction of vanillin to MMP. Excellent yields are obtained when lignin biomass derivatives are hydrogenated in the aqueous phase using the Ni/NDC catalyst that Brandi *et al.* prepared.^14^ The increased specific surface area of the nitrogen-doped carbon support is responsible for this high activity. The surface of carbon materials can be made useful by including heteroatoms in the material's skeleton.

On the one hand, heteroatoms can provide anchoring positions for active metals. At the same time, their unique electronic properties can regulate the electronic state of active metals. Heteroatoms not only directly affect grain growth but can also be used as catalyst aids.^15^ Among the above studies, nitrogen atom doping is the most representative. N atoms usually exist in various forms such as pyridine nitrogen, pyrrole nitrogen, and graphitic nitrogen.^16^ As electron donors, N atoms can strengthen the interaction between metals and carriers.^17^ Improve the dispersion of active metals and significantly improve catalytic performance.

Likewise, non-noble metals inherently have low reactivity and are limited by their resistance to deactivation and low energy efficiency caused by harsh reaction conditions during the hydrogenation of biomass platform derivatives.^18^ These catalytic systems usually require high catalyst loading, high reaction temperature, hydrogen pressure, *etc.*, which will inevitably lead to catalyst deactivation due to aggregation and leaching of metal particles.^19^ Therefore, it is very necessary to develop highly active and highly selective non-noble metal heterogeneous catalysts for the complete hydrogenation of vanillin to MMP under mild conditions.

The aforementioned single metal catalysts, such as Ni, Co, and Pd nanoparticles based on various carriers, are typically used to hydrogenate vanillin. When the synergistic effect, which includes geometric, electrical, and other interface effects is taken into account, the bimetallic catalyst performs comparably to its single nano-catalyst, outperforming it on average.^20^ According to Wang *et al.*,^21^ the Co/Ce@NC catalyst's hydrogenation activity can be enhanced by the introduction of Co *in situ* and CeO nanoparticles, which can also encourage mutual dispersion and increase the interaction between electron-rich N and electron-poor Co. CeO nanoparticles' catalytic transfer hydrogenation activity is enhanced by the doped Co species, which also creates more defective oxygen vacancies. Qiu *et al.*^22^ used imidazole-based cobalt–zinc molecular sieve framework/graphite carbon nitride composite materials as raw materials to prepare a Co–Zn bimetallic catalyst to achieve the complete conversion of vanillin to MMP. Zhang *et al.*^23^ found that Ni–Mo bimetallic catalysis can promote the dissociation of C–O bonds in vanillin and enhance the protonation hydrogenolysis ability. Regarding the selection of metal bases in bimetallic catalysts, Fe can be used as a promoter for Ni, mainly by participating in the adsorption of substrates to increase catalytic activity and by increasing the chemical stability of the catalyst to maintain catalytic activity.^24^ Kalong *et al.*^18^ adjusted the proportions of Ni and Fe in the catalyst for 5-hydroxymethylfurfural hydrogenolysis experiments and found that the addition of Ni reduced the metal load in the Fe/Al_2_O_3_ catalyst and promoted the interaction of the Fe/Al_2_O_3_ catalyst and reduction effect while increasing the acidic center of the Fe/Al_2_O_3_ catalyst.

**Fig. 1:**
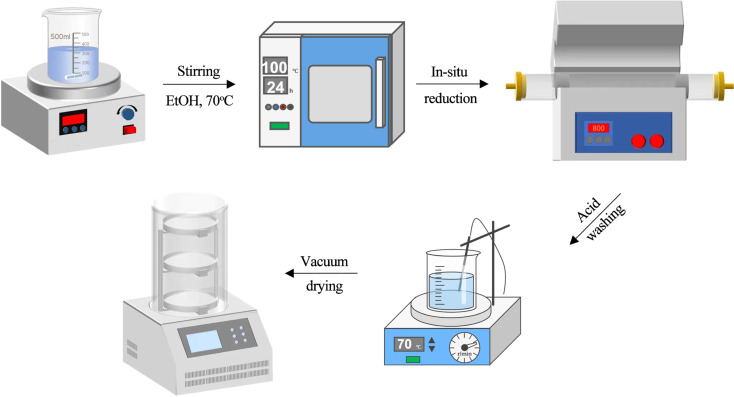
One-pot synthesis method to prepare Ni/Fe@NDC catalyst.

It is conceivable to assume that Ni and Fe bimetallic catalysts can encourage the full hydrogenation reaction of aldehydes based on the research findings mentioned above. In this work, nitrogen-doped carbon materials were prepared as supports for nickel/iron bimetallic catalysts and biomass platform compounds using the method shown in Fig. 1. The template substrate for the selective hydrogenation of vanillin to MMP is used. Bimetals and the nitrogen-doped carbon support work synergistically to produce additional active sites. The catalyst performs admirably in the catalytic hydrogenation of this reaction and is simple to separate and recover under the effect of magnetism. A continuous flow process can then be used to carry out the reaction in a micro-packed bed reactor, which can further improve the reaction conditions and forward the process of selective hydrogenation of biomass platform compounds in a more mild and environmentally friendly direction.

## 2. Experimental section

### 2.1. Chemicals

Nickel nitrate hexahydrate (Ni(NO_3_)_2_·6H_2_O, AR, 98%), ethanol (C_2_H_5_OH, AR, ≥99.7%), and methanol (CH_3_OH, AR, ≥99.5%) were purchased from Sinopharm Chemical Reagent Co., Ltd; iron nitrate nonahydrate (Fe(NO_3_)_3_·9H_2_O, AR, 98.5%), melamine (C_3_H_6_N_6_, 99%), citric acid (C_6_H_8_N_7_, 99%), isopropyl alcohol (C_3_H_7_OH, AR, ≥99.5%), *n*-decane alkane (C_10_H_22_, 98%), commercially available 10% Pd/C catalyst, and commercially available 5% Ru/C catalyst were purchased from Shanghai McLean Biochemical Co., Ltd; vanillin (C_8_H_8_O_3_, 99%), vanillyl alcohol (C_8_H_10_O_3_, 98%), 4-methylguaiacol (C_8_H_10_O_2_, >99%) were purchased from Shanghai Aladdin Chemical Technology Co., Ltd; cyclohexane (C_6_H_12_, ≥99%) was purchased from Shanghai Merrill Biochemical Technology Co., Ltd; laboratory homemade deionized water (*σ* < 5 μS m^−1^).

### 2.2. Synthesis of Ni/Fe@NDC catalyst

Dissolve nickel(ii) nitrate hexahydrate (0.03 mol), iron(iii) nitrate nonahydrate (0.01 mol), citric acid (0.03 mol), and melamine (0.03 mol) in 80 mL of absolute ethanol. The mixture was stirred and aged at 70 °C and 300 rpm for 4 h until a yellow-brown suspension was obtained and then placed in a drying oven at 100 °C overnight to remove excess solvent. After complete drying, it was ground into powder in a mortar. Then it was calcined at 800 °C for 3 hours in a tube furnace with 50 mL per min N_2_ (99.999%) airflow, and the temperature rise rate was controlled at 5 °C min^−1^. The calcined black solid powder was placed in 1 mol per L H_2_SO_4_ at 70 °C, and the solution was observed to gradually turn green, to remove unloaded metal particles. Wash with deionized water several times until the waste liquid becomes neutral, freeze it in a −89 °C freeze dryer, and obtain the Ni/Fe@NDC catalyst after complete drying.

### 2.3. Characterization of the catalysts

Catalyst morphology was observed using scanning electron microscopy (SEM), which was performed on an analytical SEM SU-70 (Hitachi, Japan) microscope operating at a voltage of 10 kV. Catalyst surface loading was analyzed using transmission electron microscopy (TEM), which was performed on a JEM-2100F microscope (JEOL, Japan) operating at a voltage of 200 kV. Use X-ray diffraction (XRD) to scan and analyze the prepared catalyst powder to obtain the diffraction pattern, using Cu Kα radiation, a voltage of 40 kV, a current of 20 mA (*λ* = 1.5406 Å), and a conventional angle of 5–80°, and compared to the International Center for Diffraction Data (ICDD) powder diffraction file (PDF) database for data processing. An IRTracer 100 (Shimadzu, Japan) Fourier Infrared Spectrometer (FT-IR) was used to evaluate the possible presence of functional groups in the samples. Metal valence states were analyzed using X-ray photoelectron spectroscopy (XPS) with Al Kα X-ray radiation on an Escalab 250 Xi photoelectron spectrometer (Thermo Fisher Scientific, USA) with a scan number of 5. The Brunauer–Emmett–Teller (BET) surface area and pore volume of the nanocomposites were measured by nitrogen physical adsorption at liquid nitrogen temperature on a Mac-ASAP-2460 (Quantachrome, U.S.A.). Pore size distributions were obtained using the Barrett–Joyner–Halenda (BJH) method.

### 2.4. Typical process for hydrogenation of vanillin

The selective hydrogenation experiment of vanillin was carried out in a 500 mL stainless steel autoclave. The inside of the autoclave was equipped with a metal aluminum fastener that could be put into up to six 20 mL quartz tubes for parallel experiments. In a typical experiment, the substrate (0.5 mmol), catalyst (20 mg), and solvent (5 mL) were added to the quartz tube, placed in the reaction kettle, and sealed. The reactor was purged three times with hydrogen to remove residual air in the kettle, and then it was pressurized and raised to the specified temperature for reaction. During the process, the magnetic stirrer was rotated at a constant speed of 500 rpm. After the reaction is completed and the temperature drops to room temperature, the remaining gas is discharged, the liquid product is put into a sampling bottle, and the reaction product is analyzed by a gas chromatograph (GC).

The liquid phase products were analyzed using a GC-2014C (Shimadzu, Japan) equipped with a capillary chromatography column (HP-5, 30 m × 250 mm × 0.25 μm) and a flame ionization detector (FID). The temperature program is set as follows: the injection port is 280 °C, the column temperature increases from 80 to 280 °C at a heating rate of 10 °C min^−1^ and is maintained for 5 min, and the detector temperature is 300 °C. Use *n*-decane as the internal standard for quantitative analysis of the product. The conversion rate and selectivity are calculated as follows:Conversion (%) = (1 − *W*_feedstock_/*W*_feedstock_) × 100%Selectivity (%) = *W*_single product_/∑*W*_single product_ × 100%

## 3. Result and discussion

### 3.1. Catalysts characterization

**Fig. 2:**
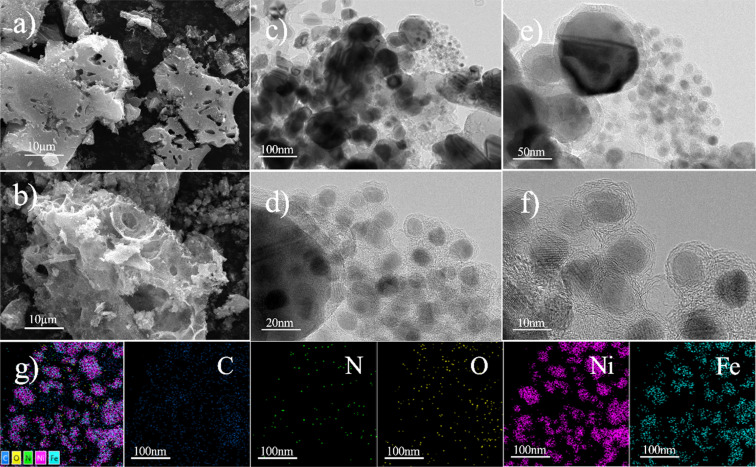
SEM (a and b) and TEM (c–f) images, and the corresponding elemental mapping (g) images of the Ni/Fe@NDC catalysts.

The Fig. 2 shows the surface morphology of the nitrogen-doped graphene-supported Ni/Fe bimetallic catalyst. Through SEM (Fig. 2a and b), it can be visually observed that the surface of the Ni/Fe@NDC catalyst has an obvious pore structure and the metal particles are dispersed evenly. Through TEM (Fig. 2c–f), it can be observed that the water-stained circle layer is a carrier structure coated with a thin graphene layer, which can effectively isolate Ni and Fe nanoparticles from the external environment, protect metal activity and prevent metal nanoparticles from hydrogenation reactions and medium loss, making it less susceptible to oxidation and improving catalyst stability. The bimetallic nanoparticles are uniformly dispersed and covered by a carbon shell. The prepared nitrogen-doped Ni/Fe@NDC catalyst is encapsulated by five graphene layers, which stabilizes the metal solid inside. The EDS-mapping image (Fig. 2g) further shows that the C, N, O, Ni, and Fe elements in the Ni/Fe@NDC catalyst prepared by the *in situ* synthesis method exist and are evenly distributed.

**Fig. 3:**
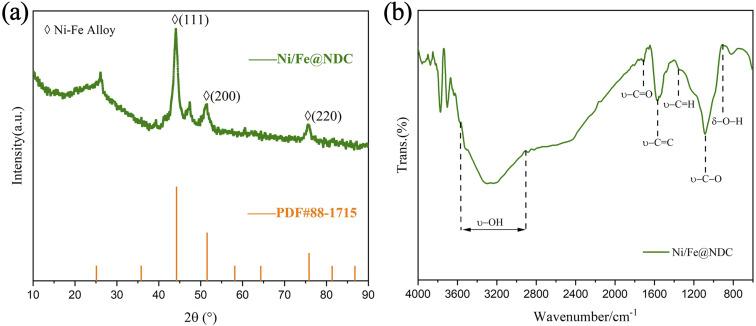
XRD (a) and FT-IR (b) images of Ni/Fe@NDC catalyst.

The crystallinity and phase information of the carbonaceous Ni/Fe@NDC catalyst material were investigated using XRD. The XRD pattern data displayed in Fig. 3a indicate the presence of a peak located between 20° and 30°. The results reported are consistent, and the graphitic carbon shell C (002) confirms that a thin graphene shell has developed in the catalyst, which is compatible with earlier research.^25^ The diffraction peaks at 2*θ* = 44.216, 51.516, and 75.842 angles were compared and found to belong to the (111), (200), and (220) crystal planes of the Ni–Fe alloy (PDF#88-1715), respectively. Indicating that Ni and the Fe bimetallic alloy has been wrapped in a graphene shell. FT-IR spectroscopy revealed acid functional groups on the Ni/Fe@NDC catalysts (Fig. 3b), where the characteristic peaks of hydroxyl and carboxyl groups indicate that there is a sufficient amount of acid sites in the catalytic process, and that the introduction of metallic substances could act as moderately acidic Lewis sites to facilitate the possible detachment of the C–O bonds.

**Fig. 4:**
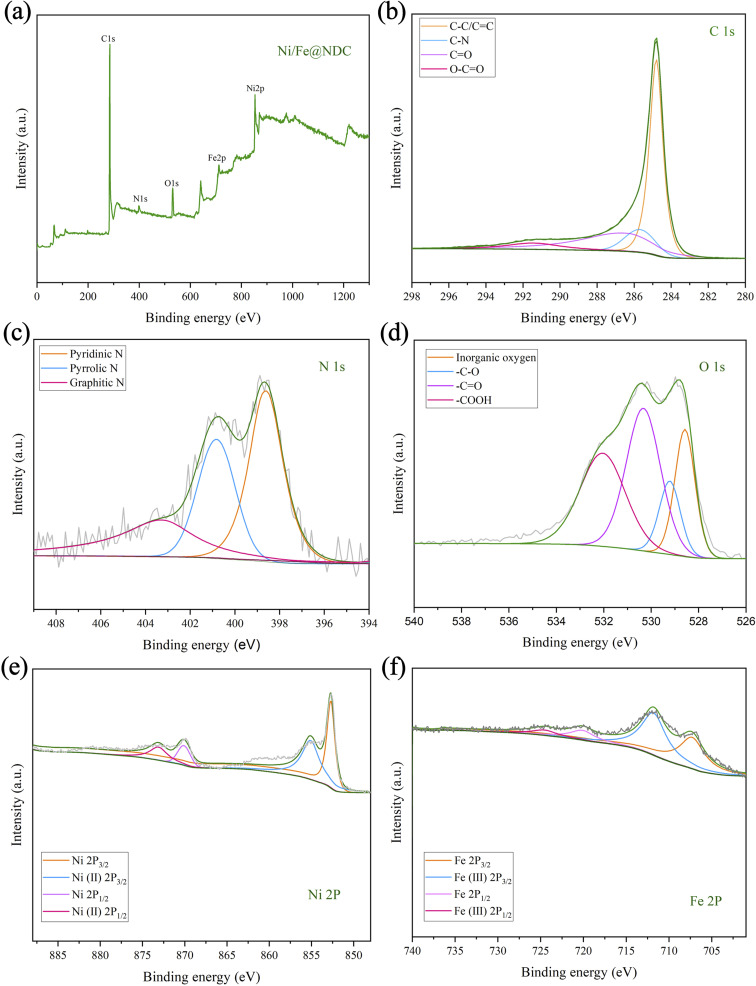
XPS (a–f) images of Ni/Fe@NDC catalyst.

Subsequently, XPS was further used to analyze the valence state of the metal elements in the Ni/Fe@NDC catalyst, the chemical composition of the surface, and the interaction of the interface (Fig. 4a–f). It can be seen from the C 1s energy spectrum that the important bond of C–C/C=C at 284.80 eV reflects the presence of a graphene structure covering metal atoms in the catalyst. On the other hand, the peak position can also be seen intuitively from the N 1s orbital diagram. They are 398.60 eV, 400.82 eV and 403.27 eV respectively. Looking at the nitrogen atom binding energy range, it is not difficult to see the formation of chemical bonds of pyridine nitrogen, pyrrole nitrogen and graphite nitrogen respectively. From the XPS peak, the atomic content of N is 3.77%. It can be seen that N atoms have been successfully doped into the graphite shell. Based on the O 1s spectrogram, it can be visualized that the first peak appears at 528.58 eV. This position represents the metal oxide network, while the subsequent peaks at 529.28 eV (–C–O), 530.38 eV (–C=O), and 532.08 eV (–COOH) are related to the presence of hydroxyl and carboxyl groups in the inner surface. In addition, the binding energies of 852.7 eV and 870.09 eV are attributed to the Ni^0^ species, and the binding energies of 855.10 eV and 873.10 eV belong to the Ni^2+^ species. The presence of zero-valent metal in the catalyst indicates the successful loading and reduction of Ni. Likewise, binding energies of 707.27 eV and 720.10 eV can be identified as Fe^0^ species, while binding energies of 711.80 eV and 724.53 eV can be identified as Fe^3+^ species. The metal presents a zero-valence state and a positive valence state in the catalyst, with the zero valence state occupying a dominant position in the catalyst, thereby achieving catalytic reduction of the substrate.

**Fig. 5:**
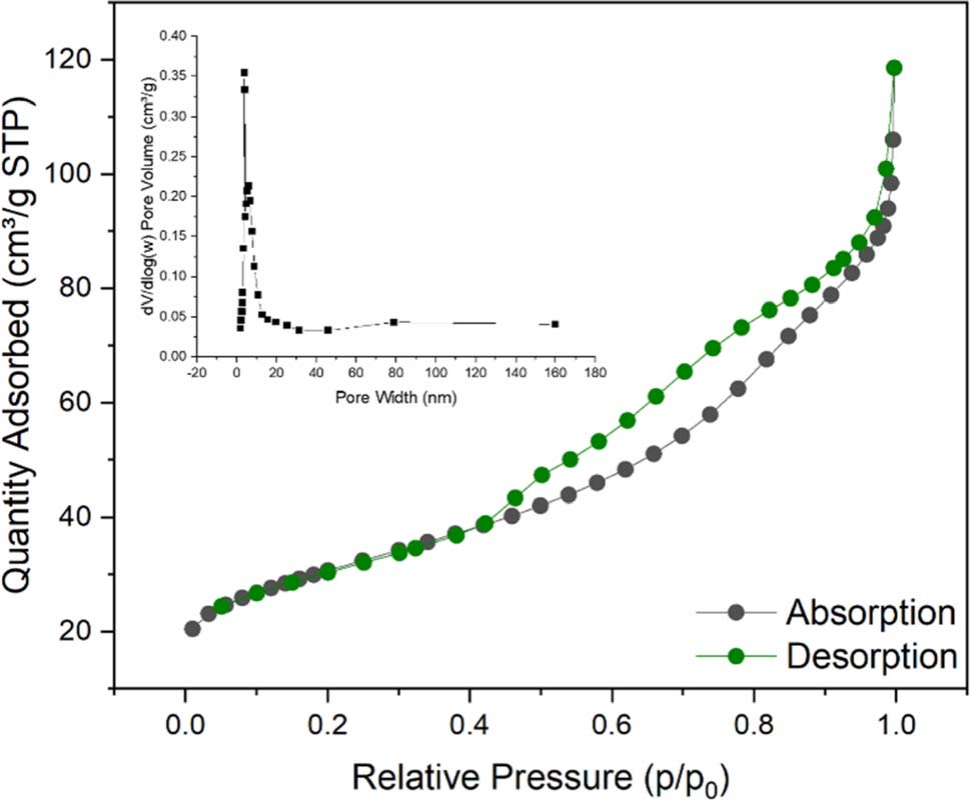
BET image of Ni/Fe@NDC catalyst.

According to the N_2_ adsorption–desorption isotherm curve shown in Fig. 5, it can be confirmed that the Ni/Fe@NDC catalyst has formed a porous structure, with a specific surface area of 107.602 m^2^ g^−1^ and a pore of approximately 0.158 cm^3^ g^−1^. According to the result that the average pore size is about 6.931 nm, it can be seen that the pore is a mesoporous structure with a regular structure (Table 1). A hysteresis phenomenon was observed in the test interval, forming an H3-type hysteresis loop, which also indicated that the catalyst was a solid containing narrow pores. This kind of nanoscale catalyst benefits from the small size effect, a higher surface atomic ratio, and a relatively high number of exposed reaction active sites, so it will have higher catalytic performance.

**Table 1:** Physical properties of Ni/Fe@NDC catalyst measured in BET

Catalyst	*S*_BET_ (m^2^ g^−1^)	Pore volume (cm^3^ g^−1^)	Average pore size (nm)
Ni/Fe@NDC	107.602	0.157751	6.9307

### 3.2. Catalytic activity

An *in situ* synthesis method was successfully used to synthesize Ni/Fe@NDC nitrogen-doped carbon carrier graphene-wrapped bimetallic nanoparticles, which were then used in experiments to verify the catalytic activity by selectively hydrogenating a biomass platform derivative called vanillin (Fig. 5). The commercially available noble metal-based catalysts Ru and Pd demonstrate remarkable conversion rates during the catalytic hydrogenation of vanillin; nevertheless, their respective selectivities of 87.42% and 91.57% are inferior to those of the Ni/Fe@NDC catalyst that has been calcined at 800 °C. And the selectivity of MMP is good. On the other hand, the control catalysts were also prepared by *in situ* synthesis method, and Ni/Fe@NDC-600 and Ni/Fe@NDC-700 catalysts were obtained by changing the calcination temperatures to 600 and 700 °C, respectively, based on the original catalysts, and Ni@C and Fe@C carbon-carrying catalysts based on citric acid. The Ni/NiO@C catalysts were obtained by calcination at 200 °C for 2 h in an oxidant atmosphere (O_2_/Ar, 95/5), and the nitrogen-doped carbon carrier catalysts Fe@NDC were prepared from citric acid and melamine.

**Fig. 6:**
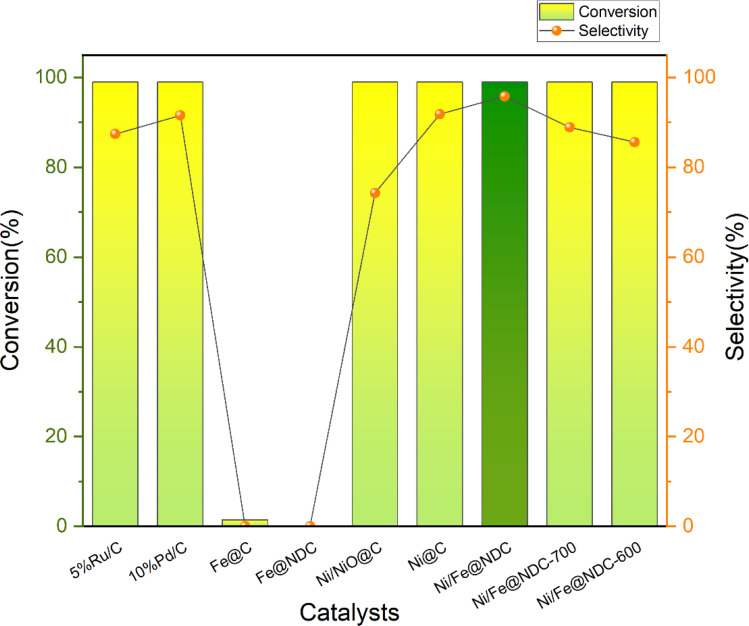
Conversion rate of vanillin and selectivity to target products on different catalysts (reaction conditions: 0.5 mmol vanillin, 5 mL isopropyl alcohol, 20 mg cat., 200 °C, 1.5 MPa, 4 h).

The efficient conversion of vanillin by the Fe element, whether supported by a carbon or nitrogen-doped carbon carrier, is negligible when it comes to the catalyst hydrogenation effect of transition metal-based synthesis. On the contrary, metal Ni exhibits remarkable potential in catalyzing the hydrogenation of vanillin, leaving nearly no substrate behind. Selectivity for the target product can be effectively changed from 91.84% to 95.83% by doping nitrogen atoms in the target product. In a similar vein, Ni/NiO did not help much in increasing the selectivity of the target product, only 74.26%. In addition, by comparing the catalysts prepared at different calcination temperatures in the tube furnace, it can be seen that increasing the calcination temperature plays a positive role in the yield of the reaction. The catalytic effect of the catalyst at 800 °C is significantly higher than that of the catalyst prepared at 600 °C. The catalyst has only 85.61% selectivity for MMP in 600 °C according to the results in Fig. 6.

**Fig. 7:**
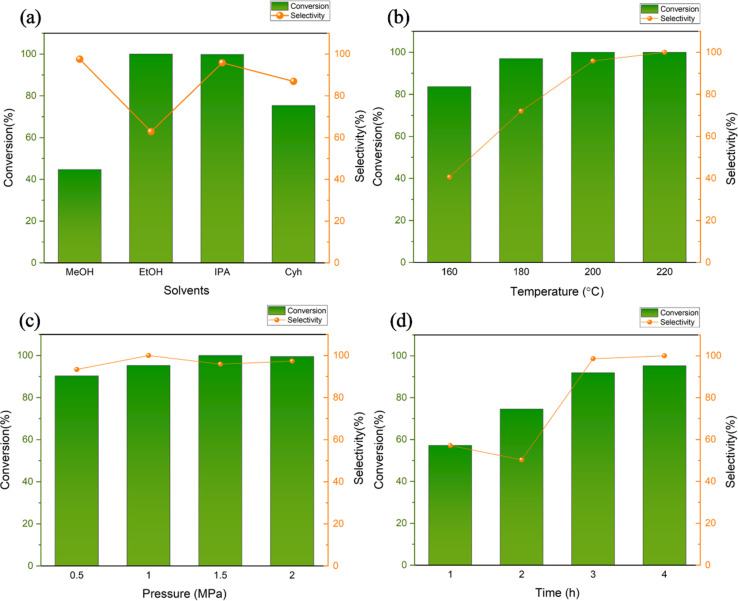
Effects of solvents, temperature, pressure and time on reactions (reaction conditions: (a) 0.5 mmol vanillin, 5 mL solvent, 20 mg cat., 200 °C, 1.5 MPa, 4 h; (b) 0.5 mmol vanillin, 5 mL isopropyl alcohol, 20 mg cat., 1.5 MPa, 4 h; (c) 0.5 mmol vanillin, 5 mL isopropyl alcohol, 20 mg cat., 200 °C, 4 h; (d) 0.5 mmol vanillin, 5 mL isopropyl alcohol, 20 mg cat., 200 °C, 1.0 MPa).

The solvent may serve as a medium and a hydrogen donor in the reduction reaction. The vanillin hydrogenation reaction was conducted at 200 °C, 1.5 MPa, for four hours, with four different solvents-methanol, ethanol, isopropyl alcohol, and cyclohexane-selected for the solvent selection process. The catalytic effect was investigated over time. Alcohols can be broadly adsorbed on the surface of metal active centers to compete for substrate molecules, as demonstrated by earlier research.^26^ This absorption ability diminishes as the length of the carbon chain increases.^27^ As an illustration, the amount of methanol absorbed is approximately twice the amount of ethanol absorbed.^28^ According to this theory, if we want to obtain a higher substrate conversion rate, we should reduce the amount of alcohol absorbed to obtain more metal-active sites. When higher carbon alcohols are used instead of methanol, the substrate conversion rate significantly increases from 44.73% (Fig. 7a). The reduction potential of alcohols also increases with the extension of the carbon chain. Primary alcohols have a higher reduction potential than secondary alcohols, which also means that secondary alcohols have stronger hydrogen donating capabilities than primary alcohols.^29^ The results from experiments show that both ethanol and isopropanol catalyze the substrate almost completely, but the selectivity of the completely hydrogenated product MMP in isopropanol is 95.83%, which is higher than the selectivity of 62.83% in ethanol. The aforementioned findings indicate that isopropanol can be used as a hydrogen source to participate in the conversion process of vanillin to MMP, and may promote the reaction path through surface acidic sites. Therefore, isopropanol is used as the reaction solvent in subsequent experiments.

Reaction temperature and pressure also have a great influence on the catalytic hydrogenation of vanillin. Compared with the intermediate hydrogenation product VA, which can be produced at a lower temperature, the synthesis of MMP is usually carried out at a temperature higher than a certain level. At 160 °C, the conversion rate of vanillin is only 83.65%, and the selectivity is 40.65%, which is an extremely low yield (Fig. 7b). When the reaction temperature is increased from 160 to 180 °C, the selectivity of MMP is significantly improved to 97.03%, the conversion rate also increases to 72.02%. In this temperature range, it is reasonable to speculate that it is the main production temperature of the intermediate VA, which means that the complete hydrogenation of vanillin to MMP is much more difficult than the hydrogenation to generate VA, and a higher reaction temperature is required to overcome the energy barrier. When the temperature reaches above 200 °C, the substrate is nearly completely converted, and the selectivity of MMP is also almost complete.

As a hydrogen donor, isopropyl alcohol can dissolve vanillin within a certain limit, promote contact between the substrate and the catalyst, and provide part of the H used in the hydrogenation reaction.^30^ Therefore, although H_2_ is the main hydrogen donor, the use of isopropyl alcohol can help reduce external H_2_ supply requirements, the reaction does not need to be performed at high pressure.^31^ Judging from the reaction results, vanillin can maintain a high level of conversion rate and high selectivity for the product MMP between 0.5 MPa and 2 MPa (Fig. 7c). The conversion rate and selectivity can reach more than 90%, so from the perspective of energy consumption. So, 1.0 MPa can be regarded as the optimal pressure under this reaction system.

Under the above reaction conditions of 200 °C and 1.0 MPa, the effect of reaction time on the catalytic effect was also explored (Fig. 7d). In the reaction time of 1 hour, although the selectivity of the target product is not low, only half of the substrate participates in the conversion (only 57.22%) and the selectivity is 57.09%. When the time was changed from 2 h to 3 h, the conversion rate was greatly improved from 74.55% to 91.93%, and the selectivity was increased to 98.61%. Then the reaction time was expanded to 4 h, the conversion rate was more than 95%, and the hydrogenation products were all MMP. Considering the catalytic efficiency, economy, and energy consumption of the catalyst, the optimal reaction system is 0.5 mmol vanillin, 200 °C, 1.0 MPa, reaction time of 4 h.

**Fig. 8:**
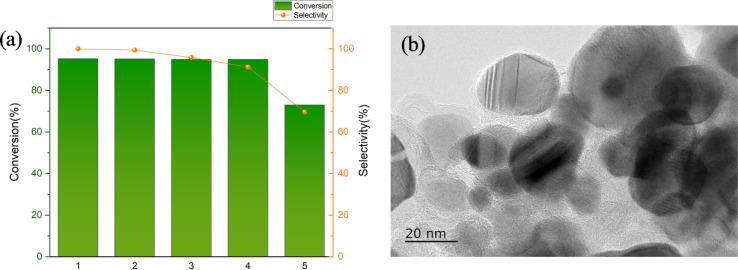
The recycling effect of the catalyst (a) and the TEM characterization test after recycling (b) (reaction conditions: 0.5 mmol vanillin, 5 mL isopropyl alcohol, 20 mg cat., 200 °C, 1.0 MPa, 4 h).

We continued to reuse Ni/Fe@NDC in vanillin hydrogenation to test its catalytic performance (Fig. 8a). Specifically, five experiments were conducted using this catalyst. The first four times the catalyst could maintain a conversion rate of about 95% while maintaining a high conversion rate of the target product. The selectivity (>90%) can prove its stable catalytic performance in cycle operation. It can be seen that the catalyst has good stability and recycling performance and can be used in the field of selective hydrogenation of aldehydes. A TEM characterization test was conducted on the recovered catalyst, and the support structure was still clearly visible (Fig. 8b). In terms of catalytic performance, a slight decrease in the conversion of the target product after the fifth reaction may be attributed to the recovery of the catalyst from the reaction mixture with the help of external magnets followed by alcohol and water washing, which is then used in the next reaction, with unavoidable losses during the washing process, leading to a corresponding reduction in the amount of catalyst and possible leaching of some of the metals leading to a decrease in the MMP yield.

### 3.3. Proposed mechanism of Ni/Fe@NDC catalyzed hydrogenation of vanillin

**Fig. 9:**
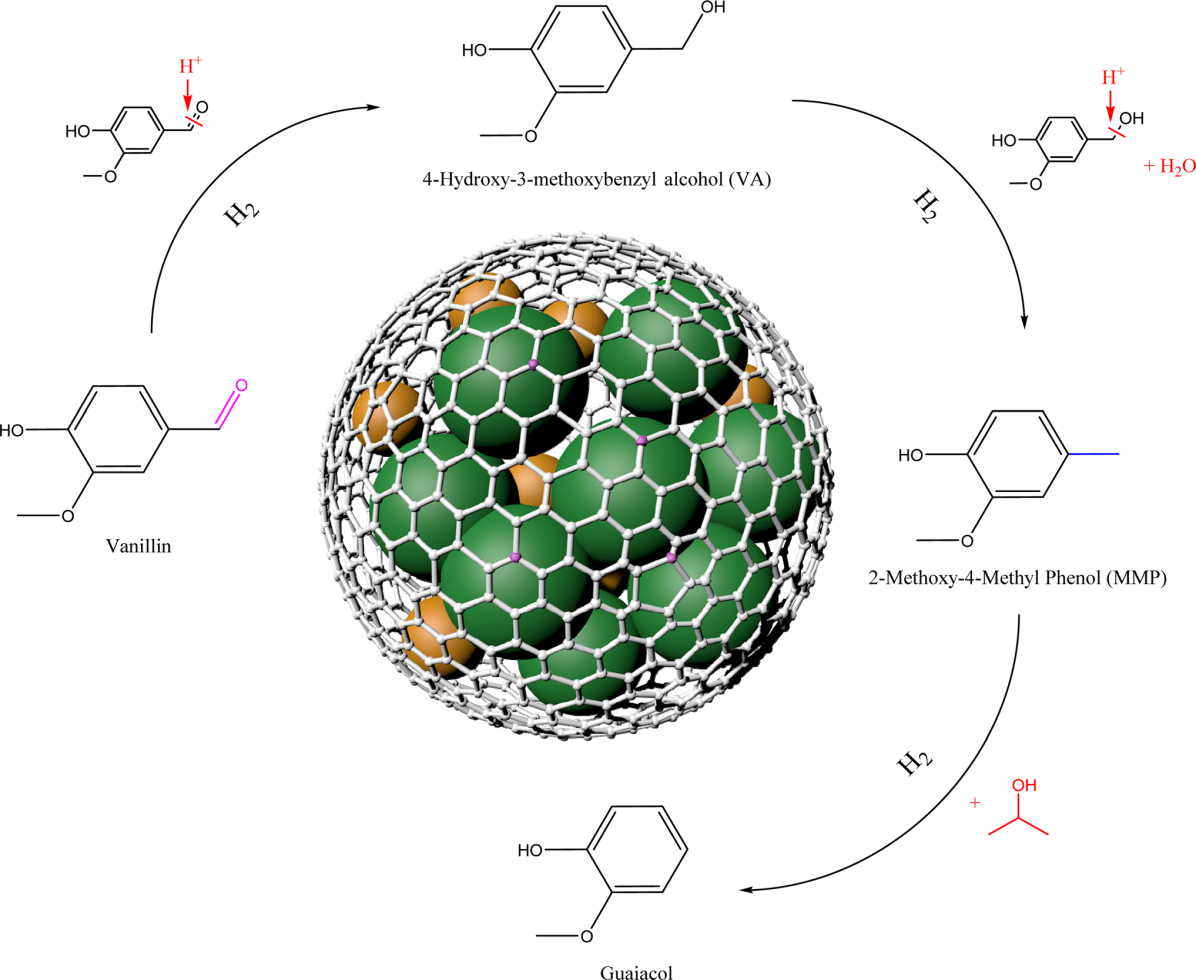
Catalytic mechanism diagram of selective hydrogenation of vanillin to prepare MMP.

Based on the above catalyst structural characterization and catalytic experimental results, a possible mechanism for the catalytic hydrogenation reaction of vanillin is tentatively proposed (Fig. 9). First, vanillin can be selectively hydrogenated to generate VA at lower temperatures. The Lewis acid center on the catalyst can activate the carbonyl group in vanillin.^32^ At the same time, adjacent Lewis acid centers adsorb isopropanol through –O on the hydroxyl group, and the metal dissociates hydrogen molecules to form hydrogen species,^33^ and then the weakened C=O bond in the vanillin molecule is attacked to break it to obtain the intermediate product VA. As the metal dispersion on the catalyst surface increases, the number of exposed metal active centers will increase. The doping of N atoms will increase the N vacancies in the carrier, which is beneficial to the adsorption and hydrogenation of C=O bonds, and the acidic center's increase is beneficial to the hydrogenolysis reaction.^34^ As the temperature conditions increase, the reaction proceeds in the direction of hydrogenolysis. The active hydrogen species combines with the hydroxyl group (–OH) in the intermediate product and removes H_2_O. Finally, the H species is connected to the methyl cation to obtain the target product MMP. Hydrogenolysis is a relatively slow process. In the early stage when the temperature is relatively low, VA remains highly selective in the product. Under higher temperature and pressure conditions, MMP will continue to transform into guaiacol,^35^ this is also worth discussing in the subsequent high-value utilization.

## 4. Conclusion

The hydrogenation of vanillin is catalyzed by a nitrogen-doped carbon-supported nickel and iron bimetallic catalyst we designed in this paper. MMP, a common component of bio-oil generated from lignin, is prepared under moderate conditions using Ni/Fe@NDC in an *in situ* synthesis approach to achieve high-value utilization. Melamine is doped into graphene to create active centers for catalytic processes and utilized as a supply of nitrogen. The catalyst has strong catalytic activity and is simple to synthesis. It has metallic magnetism and can be easily separated from the reaction product, and its repeated use has been verified through cycle tests. In particular, under the reaction conditions of 20 mg Ni/Fe@NDC catalyst, 1.0 MPa, 200 °C, and 4 h, the conversion rate of product MMP in the solvent isopropyl alcohol is 95.26%, at the same time the selectivity can reach 99%, truly realizing the selectivity of biomass platform compounds. Hydrogenation to prepare high-value chemicals has absolute reference value in batch reactions and lays a good foundation for subsequent continuous flow production.

## Author contributions

Jianguo Liu: supervise, design the research, review, and correct the manuscript. Siyi Mi: design the research, perform all experiments, preparing data and prepare the original manuscript. All authors discussed the results and assisted during manuscript preparation.

## Conflicts of interest

The authors declare no competing financial interests.

## Supplementary Material
